# Design and Implementation of a Gesture-Aided E-Learning Platform

**DOI:** 10.3390/s21238042

**Published:** 2021-12-01

**Authors:** Wolfgang Kremser, Stefan Kranzinger, Severin Bernhart

**Affiliations:** Salzburg Research Forschungsgesellschaft m.b.H., Jakob-Haringer-Straße 5, 5020 Salzburg, Austria; stefan.kranzinger@salzburgresearch.at (S.K.); severin.bernhart@salzburgresearch.at (S.B.)

**Keywords:** gesture-aided learning, e-learning, software engineering, system design, gesture recognition, system usability scale

## Abstract

In gesture-aided learning (GAL), learners perform specific body gestures while rehearsing the associated learning content. Although this form of embodiment has been shown to benefit learning outcomes, it has not yet been incorporated into e-learning. This work presents a generic system design for an online GAL platform. It is comprised of five modules for planning, administering, and monitoring remote GAL lessons. To validate the proposed design, a reference implementation for word learning was demonstrated in a field test. 19 participants independently took a predefined online GAL lesson and rated their experience on the System Usability Scale and a supplemental questionnaire. To monitor the correct gesture execution, the reference implementation recorded the participants’ webcam feeds and uploaded them to the instructor for review. The results from the field test show that the reference implementation is capable of delivering an e-learning experience with GAL elements. Designers of e-learning platforms may use the proposed design to include GAL in their applications. Beyond its original purpose in education, the platform is also useful to collect and annotate gesture data.

## 1. Introduction

The COVID-19 pandemic demanded a variety of adaptations in our daily lives, including in matters of education. With physical locations for learning and teaching closed, remote electronic educational technology (e-learning) substituted presence teaching in large parts of the world [1,2]. It is more important than ever to improve and innovate e-learning to provide better and more fruitful learning experiences.

E-learning and respective platforms have already been broadly discussed in academia. In their literature review, Arkorful & Abaidoo [1] summarize e-learning’s main advantages. Their results—which rely in large part on the work of Holmes & Gardner [2]—underline that e-learning provides (i) more flexibility in time and space for learners, (ii) increases the accessibility of knowledge, and (iii) reduces communication barriers by facilitating discussion forums. In particular, learners’ individual learning speeds are better accommodated as they can progress at their own pace [1]. For teachers, e-learning helps to overcome scarcities of teaching equipment [1]. Maatuk et al. [3] describe the challenges that come with implementing e-learning at a university level. They mention the technical and financial requirements for both providers and learners. Moreover, they find that the technological savviness of students influences the learning outcome. E-learning platforms also need to consider copyright protection and require professional development [3]. Overall they find that students are positively disposed towards e-learning and that they think that it improves their learning experience [3]. The basic building blocks of any e-learning experience are *learning objects*, i.e., the digital files that generate e-learning activities [4,5]. Learning objects come in a variety of digital formats, including e-books, 2D and 3D animations, videos, quizzes, lecture notes and presentation slides, as well as (web) applications for simulation [6].

This work takes a look at how to combine learning objects with body gestures. Learning with gestures is a well-analyzed phenomenon and can be summarized under the term *gesture-aided learning* (GAL). Novack & Goldin-Meadow [7] distinguish GAL literature into two threads: The effect of learners themselves gesturing during learning, and the effect of learners seeing instructors gesturing during teaching. Regarding the first thread, the findings of Saltz & Donnenwerth-Nolan [8] show that gestures as a form of motoric enactment help to memorize and later recall a sentence, compared to exclusively verbal-based learning. Goldin-Meadow et al. [9] find that instructing children to gesticulate while learning to solve mathematical problems results in a better learning outcome compared to a control group in which children do not gesture. Interestingly, the authors find that it is important for the learning outcome that gestures are executed “correctly”. Novack & Goldin-Meadow [7] analyzed the relevant literature and offer four explanations on how GAL might result in better learning outcomes. First, gestures can help to link abstract concepts to the immediate environment [10]. Second, gesturing during the learning process can reduce an individual’s cognitive load [11,12,13,14,15]. The third explanation argues that spoken communication is enhanced by gesturing [16]. Finally, it discusses findings in the literature that emphasize that gesturing enhances learning because it can engage [17] or activate [18] the motor system. Recent findings by Scheiter et al. [19] suggest that motoric enactment works best if the learning task is of medium difficulty and the gesture to enact is clearly defined.

With respect to the second thread in GAL literature, which covers how seeing a gesture enhances learning, Tellier [20] shows that schoolchildren achieve better results in learning a foreign language when teachers incorporate gestures in the teaching process. Macedonia & Kriegstein [21] provide a comprehensive literature review on how gestures improve performance in foreign language learning. They find that gestures can provoke and enhance the sensorimotor representation of a word or sentence and thereby improve the embodiment of abstract words. However, the concept of GAL is not limited to foreign language learning. Valenzeno et al. [22] show in an experiment that children achieve better results in learning the concept of symmetry when teachers use their hands while teaching this concept. The same is true when children learn to solve abstract mathematical problems [23], even if the instructor is an animated avatar [24]. The positive effect of gestures also translates to instructors that appear on video [25]. To summarize, we find results that indicate that gestures improve the learning effect when they are used by teachers and/or learners during the learning process.

Despite its positive effects on learning, to the best of our knowledge, a GAL-based e-learning system has not yet been documented. To close this gap, this work aims to answer the following research question: Can e-learning platforms facilitate gesture-aided learning remotely? To answer this question, this work proposes a system design for e-learning platforms with GAL elements (e-GAL). To demonstrate the design’s ability to facilitate GAL, we test a reference implementation. We consider the reference implementation, and thus the e-GAL design, to be successful if (a) instructors can effectively plan and monitor gesture-enhanced lessons, (b) learners are able to comprehend and imitate gestures and learning objects during lessons, and (c) learners accept the system. This study contributes to the existing literature by demonstrating how elements of GAL can be integrated into e-learning applications.

## 2. E-GAL System Design

This section proposes the e-GAL design, a system design for e-learning platforms with GAL support. It is open regarding the type of learning content and was developed specifically for this study. Central to the design is the decoupling of learning content and gestures. This way, already recorded gestures can be shared and reused between use cases. The sharing of learning objects is a common occurrence in e-learning communities [4].

### 2.1. Data Model

**Figure 1:**
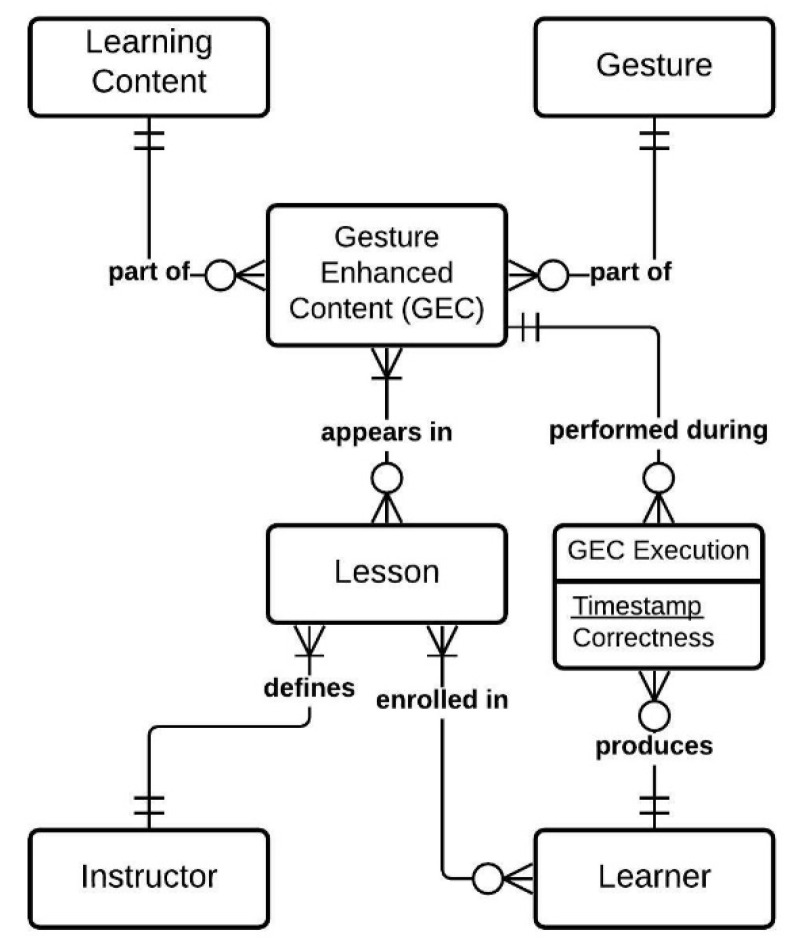
The entity-relationship diagram of the e-GAL data model in crow’s foot notation.

While the learning content itself remains in its original form (e.g., text, sound, video), it is communicated with the addition of a gesture. This gesture does not replace, but rather enhances the learning content. Thus, we call this combination of learning content and gesture *gesture-enhanced content* (GEC). Consequently, a *lesson* is an ordered list of GECs. The *instructor* is responsible for defining GECs and lessons, while the *learner* executes the lesson by performing all GECs within it and creating *GEC executions* by doing so. The resulting data model is depicted in Figure 1.

### 2.2. Modules

**Figure 2:**
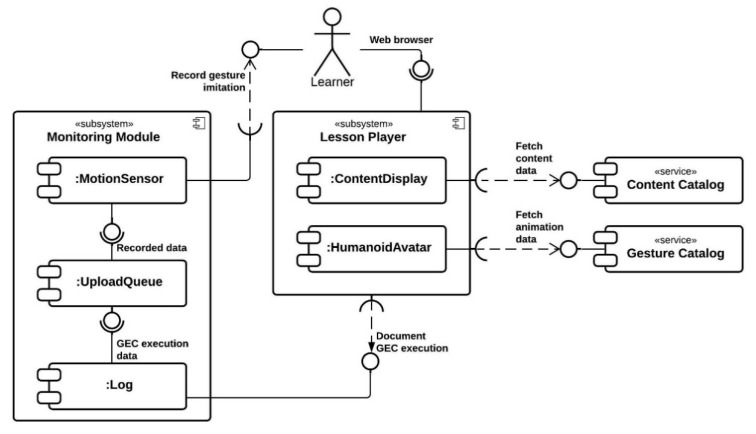
UML component diagram from the perspective of the learner.

**Figure 3:**
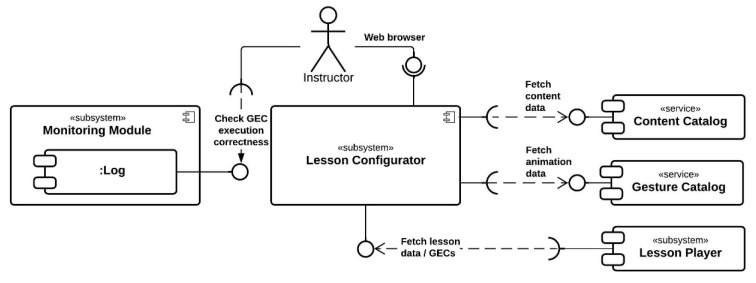
UML component diagram from the perspective of the instructor.

The e-GAL design is composed of 5 modules. Figure 2 and Figure 3 illustrate how the modules are connected and how learners and instructors respectively are supposed to interact with the system.

#### 2.2.1. Content Catalog

The content catalog is a database that holds learning content items in one or multiple formats (e.g., text, audio, video). If the content items are large in size, the catalog should carry file references rather than the actual learning content data, or a database management system that supports large fields should be used to avoid performance issues.

#### 2.2.2. Gesture Catalog

The second database is the gesture catalog. It holds pre-recorded reference gestures in one or more file formats, which may vary depending on what was used to record the gestures. However, the gesture data must be sufficient to animate a humanoid avatar (see lesson player module). Ideally, the gestures are recorded with a high-quality motion-capture system to produce the best possible reference.

#### 2.2.3. Lesson Configurator

The lesson configurator is a web-based service with a graphical user interface (GUI) for instructors that allows them to combine learning content items and gestures into individual GECs. Multiple GECs can be organized into lessons, and additional lesson parameters (e.g., lesson speed) can be set.

#### 2.2.4. Lesson Player

Learners interact with the platform via the web-based lesson player. It replays GECs by depicting a humanoid avatar alongside content items. The avatar is animated using the gesture reference data from the catalog. Alongside the gesturing avatar, one or more output ports (e.g., text display, speaker output) replay the content items.

#### 2.2.5. Monitoring Module

As mentioned in the introduction, research indicates that gestures need to be performed correctly for GAL to provide benefits [9]. The monitoring module records motion data using some type of sensor (e.g., accelerometer, video) and transfers them to the instructor for review. The choice of motion sensor depends on the gesture’s range of motion. For instance, if gestures are only performed with hands, a wrist-mounted inertial measurement unit might suffice to retrace the performed gesture [26]. Full-body gestures on the other hand may require a more complex measurement setup. The recorded motion data, along with metadata about the learner and the performed gesture, get uploaded into the log. The log holds all data about past GEC executions and provides an interface for the instructor to look at the motion data and assess the correctness of the gestures.

## 3. Materials and Methods

### 3.1. Reference Implementation

We demonstrated and evaluated a reference implementation of the proposed e-GAL system design (see Section 2). The learning task of this reference implementation was to learn a series of German language words. The design’s modules were deployed in a microservice pattern [27] and implemented as follows.

The content catalog consisted of 64 German language words which is a subset of the words used in Mathias et al. [28]. In addition to the textual representation, synthesized speech by Google’s WaveNet-based text-to-speech engine [29] was added. Both text and speech were stored in a PostgreSQL 13 database [30].

For each word, a representative gesture (cf. [28]) was recorded using the full-body motion-capture system XSENS MTw Awinda [31]. After recording, each gesture was exported into an FBX file to be suitable for animating the avatar. The FBX file reference for each gesture was stored in the gesture catalog database.

**Figure 4:**
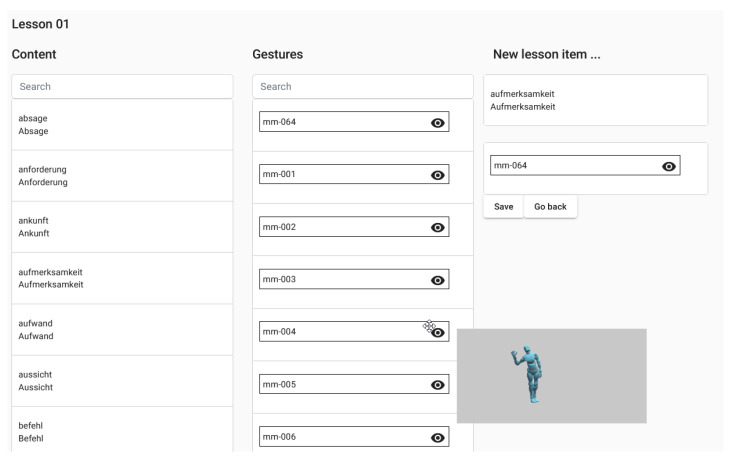
Screenshot of the reference implementation’s lesson configurator. GECs could be created by drag-and-drop of the content and gesture cards. Hovering over a gesture card previewed the gesture.

In the implementation of the lesson configurator (Figure 4), instructors could combine a word with a gesture by drag-and-drop in their browser. An important feature was the ability to preview gestures on the fly since labels were rarely sufficient for describing what a gesture looked like. Available lesson parameters included the lesson speed, i.e., the time between two GECs, and a randomization seed with which the order of GECs was shuffled. Furthermore, instructors could generate individualized hyperlinks with which students could start the lesson.

**Figure 5:**
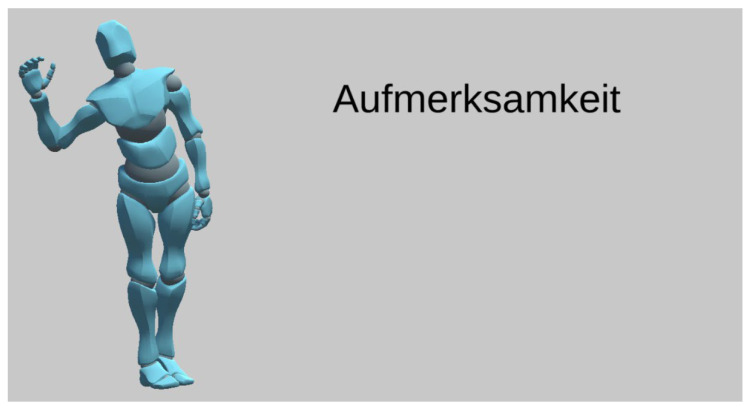
Screenshot of the reference implementation’s lesson player. A robot-like avatar enacted the gesture, while the learning content was displayed in text and spoken out loud by the text-to-speech engine.

The lesson player (Figure 5) of the reference implementation was a Unity 3D [32] application running in a WebGL environment. It featured a robot-like avatar (“Y-Bot” [33]) on a neutral background. When the learner started the lesson, the Unity application was loaded alongside the necessary lesson data, namely the learning content items and the gestures’ FBX files. After loading, the lesson player played each GEC one after the other by simultaneously displaying the word and playing the sound clip (see Video S1). Then, after a small delay, the avatar performed the gesture. This was repeated for each GEC until the lesson was completed.

**Figure 6:**
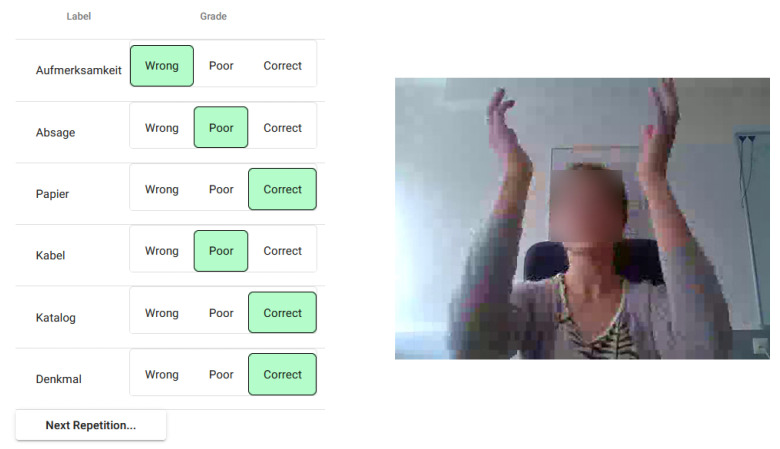
The instructor’s web interface for reviewing and labeling GEC executions.

It was assumed that the learner sits behind their desk and in front of their screen during learning. Their computer’s webcam, therefore, was most likely to capture at least the upper body. During each GEC, the participant was recorded and after each GEC, the recorded video clip was annotated with the GEC execution ID and queued up for upload to the monitoring modules log. The instructor could access and rate the videos in the monitoring module’s web interface (Figure 6).

### 3.2. Evaluation of Reference Implementation

A system test was conducted to assess the e-GAL reference implementation’s capability to facilitate remote GAL. We want to note that we do not claim to measure actual learning progress, as this would require more sophisticated methods from other fields closer to neurology. Rather, this study aims to answer the research question of whether e-learning can deliver GAL, and in the course validating the proposed e-GAL design.

**Participant recruitment:** 20 people were recruited by email for the system test. Each participant received an individualized link that allowed them to take the prepared lesson at any time and place during the 2-week trial period in July 2021. One person could not finish the experiment due to technical difficulties with the web application. Ultimately, we used data from 12 female and 7 male participants with a mean age of 36.6 (σ = 9) ranging from 23 to 53 years. The majority of the participants worked in technical affine companies, therefore, a basic knowledge of using web applications was assumed. Each participant gave their informed consent to be recorded before starting the experiment.

**Experiment design:** An instance of the reference implementation (see Section 3.1) was made accessible online. The authors, acting as instructors, created a lesson containing six GEC items with the lesson configurator. Video S1 in the Supplementary Material contains a screen recording of the full lesson. The gestures were chosen based on whether they could be performed while sitting behind a desk. The GECs order was randomized, with the same randomization seed for each participant.

Participants could access the lesson via their personal invitation link. After displaying the informed consent form and instructions, participants had the chance to preview their webcam feed to make sure they were comfortable with what was being recorded. After accepting, the lesson player started in their web browser. The lesson started shortly after, and one GEC after another was played. Between GECs was a break of three seconds, during which participants were supposed to imitate the avatar while reading the displayed word out loud. The webcam started recording when a new GEC was played and ended 2.5 seconds later. The videos were stored locally and queued up immediately for background upload to the monitoring module’s log.

After all GECs of the lesson had been repeated 4 times, the player stopped and redirected the participant’s browser to a German translation of the SUS, originally introduced by Brooke [34] and translated by Reinhardt [35]. The SUS questionnaire is comprised of the ten items in Appendix A Table A1, which are answered with a five-point Likert scale [36] ranging from “strongly disagree” to “strongly agree”. To calculate the SUS, each response option gets scored from one to five points, starting with one point for “strongly disagree” to five points for “strongly agree”. The next step is to adjust the points of the questions. For all odd questions, we subtract one point and for all even questions, we subtract the value five from their score. Next, we add up the points for each of the ten questions and multiply this sum by 2.5. Finally, we get a usability score for each respondent, ranging from 0 (worst) to 100 (best).

Afterward, the participants filled in a questionnaire that asked them about their remote lesson experience (RLE). The questionnaire contained five items which are presented in Appendix B Table A2 and could be answered with a five-point Likert scale [36] ranging from “strongly disagree” to “strongly agree”. These questions were supposed to identify any problems in the presentation of the learning content or gestures. For evaluation, we subtract a value of one from each question. Next, we take the mean over all participants per question and get a score ranging from 0 (strongly disagree) to 4 (strongly agree). Additionally, an open question allowed participants to freely comment on their thoughts regarding the platform.

**Interpretation of the results:** To assess the learners’ acceptance of the system, we follow Bangor et al. [37,38] and use three different rating scales for interpreting the SUS results.

*Adjective rating*: According to Bangor et al. [37,38], the SUS score can be converted into an adjective rating to interpret its results. They show that the results of a seven-point Likert scale correlate with SUS scores and can therefore be useful for interpretation. The findings of Bangor et al. [37] show that the SUS score has a mean of 12.5 when using the adjective “Worst Imaginable” to describe a system, 20.3 when using “Awful”, 35.7 when using “Poor”, 50.9 when using “Ok”, 71.4 when using “good”, 85.5 when using “Excellent” and 90.9 when using “Best Imaginable”. Except for “Worst Imaginable” and “Awful”, all of these adjectives are significantly different and are therefore of interest for the interpretation of the results. e.g., if the SUS score is 75, we would classify our platform as “Good”.

*Grade scale*: Bangor et al. [37] introduce the so-called university grade analog, in which the SUS scores are related to school/university grades. According to this grading scale a SUS score between 90 and 100 is an A, 80 and below 90 is a B, 70 and below 80 is a C, 60 and below 70 is a D, and a score below 60 is an F.

*Acceptability rating*: Moreover, to decide whether the platform is usable or suitable to provide GAL, we follow Bangor et al. [37,38] and use the acceptance ranges they provide. The authors rate a system with a SUS score below 50 as “Not Acceptable” and above 70 as “Acceptable”. Between a score of 50 and 70, Bangor et al. [37,38] state that the system should be improved and evaluated as “Marginal”. This group can be further divided into “Low Marginal” (SUS score between 50 and 62.6) and “High Marginal” (SUS score between 62.6 and 70).

In sum, the adjective rating, grade scale, and the acceptability rating are suitable to answer the question of whether learners accept the e-GAL reference implementation.

Regarding the RLE responses, we consider an average of 3.0 to be sufficient. At this level, there is general agreement that the respective lesson element was comprehensible. An exception is question 5 (“*I felt insecure during the lesson.”*) which is reverse coded to check the consistency of the participant’s answers. The optional free-text comments are mapped to concepts by means of a small-scale inductive content analysis [39].

The videos of the GEC executions are visually compared against the reference gesture by the authors. Based on the difference, the GEC executions are labeled “Correct” (no discernable difference), “Poor” (recognizable as the reference gesture, but with errors, e.g., not moving the head along with the waving hand), and “Wrong” (not recognizable as the reference gesture). Videos that failed to show the gesture clearly (e.g., because the participant was out of frame) were also labeled “Wrong”.

## 4. Results

### 4.1. System Usability Scale (SUS)

**Figure 7:**
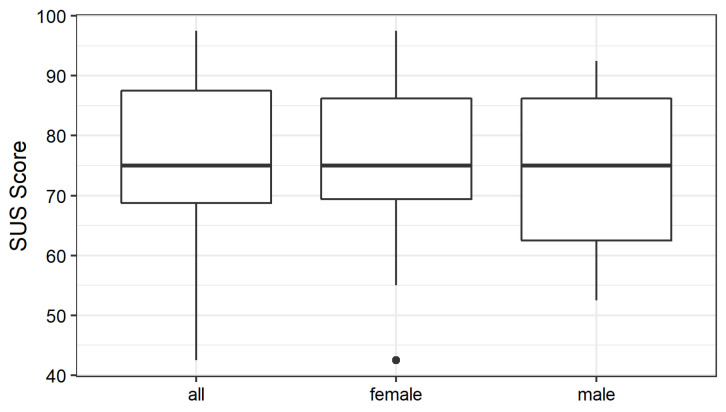
SUS score boxplots for all participants and by gender.

**Figure 8:**
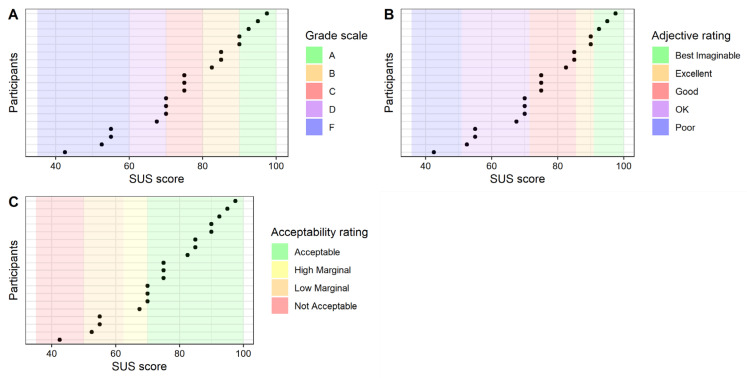
SUS score per participant: (**A**) grade scale, (**B**) adjective rating, and (**C**) acceptability rating.

Figure 7 shows boxplots for the SUS scores across all participants and female (12) and male (7) participants respectively. The median and mean SUS score was 75, with no differences between genders. Consequently, the reference implementation received a C on the grade scale, and a “Good” according to the adjective rating scale. On the acceptability rating scale, the reference implementation was rated “Acceptable”. Interestingly, the individual SUS scores varied considerably, with values between 42.5 and 97.5. Therefore, we show the results of the SUS score on the individual level to better understand the results. Figure 8 represents each participant’s SUS score located on each of the three scales: (a) shows that four out of the 19 participants rated the platform with the worst grade F (21%), one with a D (5.3%), six with a C (31.6%), three with a B (15.8%) and five with the best grade A (26.3%). When applying the adjective rating scale (b), we find that one participant rated the platform as “Poor” (5.3%), seven as “Ok” (36.8%), six as “Good” (31.6%), two as “Excellent” (10.5%), and three as “Best Imaginable” (15.8%). Finally, (c) illustrates the acceptability rating and shows that for one participant the reference implementation was “Not Acceptable” (5.3%), for three it was “Low Marginal” (15.8%), for one it was “High Marginal” (5.3%) and for fourteen it was “Acceptable” (73.7%).

### 4.2. Remote Lesson Experience (RLE)

**Figure 9:**
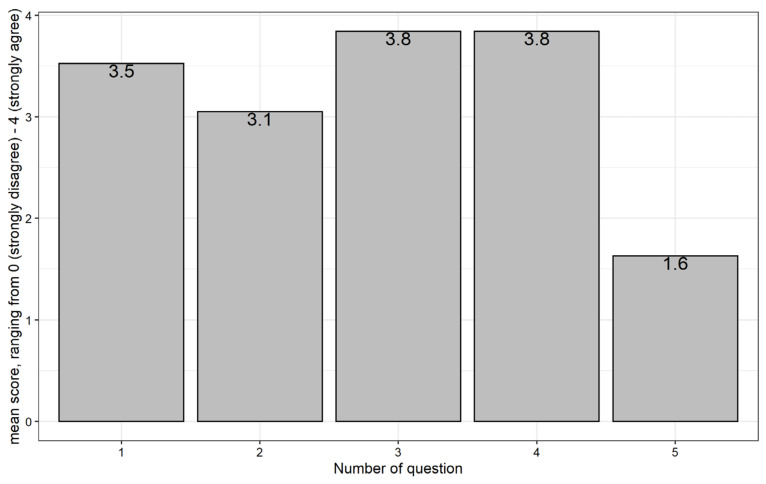
Average remote lesson experience (RLE) over all participants.

Figure 9 illustrates the results regarding the items to evaluate the RLE (Table A2). When the participants were asked if the word to learn was clearly readable and audible (Questions 3 and 4), they tended to strongly agree, with a score of 3.8 for both questions. When asked whether they were able to focus on the lesson’s content (Question 1) or whether they were able to imitate the avatar’s gestures (Question 2), we have a somewhat lower score of 3.5 and 3.1 respectively. Furthermore, with a mean value of 1.6, the participants answered that they generally did not feel insecure during the lesson (Question 5).

### 4.3. Webcam Videos

After the trial period ended, the log contained 491 GEC executions. These were more than the anticipated 456 videos. During labeling, it became apparent that some participants stopped and restarted mid-lesson. Based on these videos, 340 (69.2%) GEC executions were rated “Correct”, 95 (19.3%) were rated “Poor”, and 56 (11.4%) were rated “Bad”. The majority (54.3%) of “Poor” and “Bad” GEC executions occurred during two gestures: *“Aufmerksamkeit”* (eng.: “attention”; putting a hand behind an ear and leaning back) and *“Papier”* (eng.: “paper”; crumbling a piece of paper and throwing it away).

### 4.4. Free-Text Comments

Nine out of the nineteen participants opted to give a free-text comment about their thoughts on the lesson experience. Table 1 shows how often a concept was mentioned in the comments.

## 5. Discussion

This study set out to answer the research question: Can e-learning platforms facilitate gesture-aided learning remotely? In the case of the e-GAL reference implementation, we assume this to be confirmed if (a) instructors can effectively plan and monitor gesture-enhanced lessons, (b) learners are able to comprehend and imitate gestures and learning objects during lessons, and (c) learners accept the system.

**Ad (a):** The lesson configurator offered instructors access to 64 learning content items and 64 distinct gestures. With these materials, a lesson containing 6 GECs was successfully created. The gestures could be previewed and selected according to the assumed learning environment (i.e., the learner sitting behind a desk). Regarding lesson monitoring, the monitoring module’s log successfully collected videos of all 491 GEC executions. Instructors were able to label all videos using the monitoring module’s web interface.

**Ad (b):** Learners were able to access the lesson with the invitation link that was sent out by the instructor. In the responses to the RLE questionnaire, there was general agreement (3.8 out of 4) that the learning content was comprehensible in both text and speech. Interestingly, two participants noted that they did not use the text but rather listened exclusively to the audio. Slightly less agreed upon (3.1 out of 4) was on the comprehensibility of gestures. A possible reason for why the gesture comprehensibility (Question 2) was rated worse may be connected to the 2 least well-performed GECs. “*Aufmerksamkeit*” required the participant to lean backward, which was not easily discernible on the solid-grey background of the lesson player. A better-designed 3D environment may communicate changes in depth better. As for the second badly performed GEC, *“Papier”* involved both palms touching each other. The avatar’s extremities lacked collision boxes, therefore its hands clipped into rather than touching each other. This was interpreted differently by participants, some touching their forearms or bumping their fists. Furthermore, the XSENS skeletal model has only a rough positioning of the hands. By adding collision boxes to the avatar and including better hand sensors, the communication of gestures that feature more intricate hand movements could be improved. Broader gestures, like waving a hand, were more accurately imitated. Furthermore, the system could have better indicated the right time to imitate the gesture, especially as most of the GEC executions rated “Wrong” seemed to stem from the participant not being aware that they should imitate at that moment. In the end, 69.2% of gesture executions were labeled “Correct”.

**Ad (c):** The e-GAL reference implementation was rated “Acceptable” and “Good”, and received the letter grade C on the System Usability Scale. The evaluation of the reference implementation is limited insofar as it only considers the perspective of the learner and lacks feedback from instructors. While they were functional enough to define and monitor the experiment lesson, the lesson configurator and monitoring modules were not demonstrated in the same way the lesson player was.

To summarize, we consider all of the three requirements stated and discussed above to be fulfilled, thus we conclude the system test as successful.

During labeling, a second potential use case for the reference implementation emerged. It can collect and label large amounts of gesture data remotely and with little effort. The main issue in the video clips from the system test was that the framing of the participant was inconsistent, and their webcam quality varied. This could be solved however by better instructing the participants and by consistently checking the framing before and during the lesson.

### 5.1. Limitations

This study is concerned with the technical viability of e-GAL, thus it does not say anything about the influence of this mode of learning on learning outcomes. Claims of this kind would require a different study design and neurological evidence. Moreover, the lesson used in the study lacked pedagogical considerations (see [40]) which made it unfit to produce and measure actual learning outcomes. Finally, the evaluation of the lesson configurator lacks the perspective of educators who are not in higher education.

### 5.2. Future Work

More research on the pedagogy of e-GAL applications is needed. This includes determining the overall effectiveness of e-GAL, which parameters (e.g., repetition and order of GECs, lesson tempo) need to be adjustable, and how the avatar’s and the 3D environment’s designs affect learning outcomes. It should be investigated which types of learning content work best with e-GAL.

Future platforms could incorporate machine learning models for pose estimation (e.g., [41,42]) and/or quality assessment [43] of the performed gesture. Such automated methods could be used for example to support or replace the instructor’s subjective rating or to provide real-time feedback to the student. Furthermore, instead of the webcam as motion sensor, future systems could use wearable motion sensors to allow students more mobility. Feature requests such as avatar customization, the option to see oneself during the lesson, and immediate feedback during the lesson were mentioned by some participants. These features are realizable for the reference implementation.

Lastly, e-learning platforms usually involve a variety of stakeholders such as content creators, educational institutions, and designers [44]. The e-GAL design could be extended or embedded into existing e-learning platforms to accommodate these stakeholders (e.g., interfaces for content creators to add new gestures from other motion-capture systems). Interfaces to existing learning object repositories could produce interesting new GECs.

## 6. Conclusions

We proposed a system design for e-GAL platforms with three design goals. A reference implementation following the design was demonstrated and evaluated in a field test. After interpreting the results of the SUS & RLE, the user comments, and the number of video clips labeled “Correct”, we determined that the e-GAL reference implementation met all of the three design goals, consequently demonstrating the ability of the proposed system design to facilitate an acceptable e-GAL experience. Additionally, the reference implementation showed itself to be useful for collecting and annotating video clips of gesture executions, which can be used for instance to generate large gesture datasets for machine learning. The e-GAL design can be used to implement e-GAL applications or as the basis for further research into the topic of gesture-aided e-learning, especially its pedagogical implications.

## Figures and Tables

**Table 1:** Occurrences of concepts in the free-text comments.

Concept	# of Occurrences
Confusion on when to repeat the gesture	5
Optimism that reference implementation can improve learning	3
Missing immediate feedback during the lesson	2
Focus on spoken words instead of written words	2
Ability to see oneself during the lesson	2
Confusion about how to perform the gesture	1
Confusion about the SUS questionnaire	1
Confusion about the role of the webcam	1
Avatar customization	1

## Data Availability

Questionnaire responses are available at https://doi.org/10.17605/OSF.IO/ZCX85 (accessed on 16 November 2021).

## Supplementary Materials

The following materials are available online at https://doi.org/10.17605/OSF.IO/ZCX85, Video S1: experiment_lesson.mp4.

## Appendix A

Table A1 lists the German translation of the System Usability Score that was used for this study. While we are aware that it is not formally validated, the authors of this paper, who are German native speakers, agreed that the chosen translation is more comprehensible and unambiguous compared to the validated translation presented in [45].

**Table A1:** System Usability Scale (SUS) items.

No.	German Translation [35]	Original [34]
1	Ich denke, dass ich das System gerne häufig benutzen würde.	I think that I would like to use this system frequently.
2	Ich fand das System unnötig komplex.	I found the system unnecessarily complex.
3	Ich fand das System einfach zu benutzen.	I thought the system was easy to use
4	Ich glaube, ich würde die Hilfe einer technisch versierten Person benötigen, um das System benutzen zu können.	I think that I would need the support of a technical person to be able to use this system.
5	Ich fand, die verschiedenen Funktionen in diesem System waren gut integriert.	I found the various functions in this system were well integrated.
6	Ich denke, das System enthielt zu viele Inkonsistenzen.	I thought there was too much inconsistency in this system.
7	Ich kann mir vorstellen, dass die meisten Menschen den Umgang mit diesem System sehr schnell lernen.	I would imagine that most people would learn to use this system very quickly.
8	Ich fand das System sehr umständlich zu nutzen.	I found the system very cumbersome to use.
9	Ich fühlte mich bei der Benutzung des Systems sehr sicher.	I felt very confident using the system.
10	Ich musste eine Menge lernen, bevor ich anfangen konnte das System zu verwenden.	I needed to learn a lot of things before I could get going with this system.

## Appendix B

**Table A2:** Items for evaluating the Remote Lesson Experience (RLE).

No.	Original in German	English Translation
1	Ich konnte mich auf die Inhalte der Lektion konzentrieren.	I was able to focus on the lesson’s content.
2	Es war einfach, die Gesten des Avatars zu imitieren.	It was easy to imitate the avatar’s gestures.
3	Die gesprochenen Wörter waren klar zu verstehen.	The spoken words were clearly audible.
4	Ich konnte die angezeigten Wörter problemlos lesen.	I was able to read the displayed words clearly.
5	Ich fühlte mich unsicher während der Lektion.	I felt insecure during the lesson.
